# Supplementary material for: The protease GtgE from Salmonella exclusively targets inactive Rab GTPases
Source: Nat Commun. 2018 Jan 3;9:44. doi: 10.1038/s41467-017-02110-1 (PMC5752668; doi:10.1038/s41467-017-02110-1)
Supplement: Supplementary file 1 — Supplementary Information [file 41467_2017_2110_MOESM1_ESM.pdf]

# Supplementary Information

# The protease GtgE from Salmonella exclusively targets inactive Rab GTPases

**Table of Contents**


Supplementary Figures ........ 2
  Supplementary Figure 1 ........ 2
  Supplementary Figure 2 ........ 3
  Supplementary Figure 3 ........ 4
  Supplementary Figure 4 ........ 5
  Supplementary Figure 5 ........ 6
  Supplementary Figure 6 ........ 7
  Supplementary Figure 7 ........ 7
  Supplementary Figure 8 ........ 8
  Supplementary Figure 9 ........ 9
  Supplementary Figure 10 ........ 10
  Supplementary Figure 11 ........ 10
  Supplementary Figure 12 ........ 11
  Supplementary Figure 13 ........ 12
  Supplementary Figure 14 ........ 13
  Supplementary Figure 15 ........ 14
  Supplementary Figure 16 ........ 15
  Supplementary Figure 17 ........ 16
  Supplementary Figure 18 ........ 17
Supplementary Tables ........ 18
  Supplementary Table 1 ........ 18
  Supplementary Table 2 ........ 19
  Supplementary Table 3 ........ 20
Supplementary References ........ 21

## Supplementary Figures

### *Supplementary Figure 1*

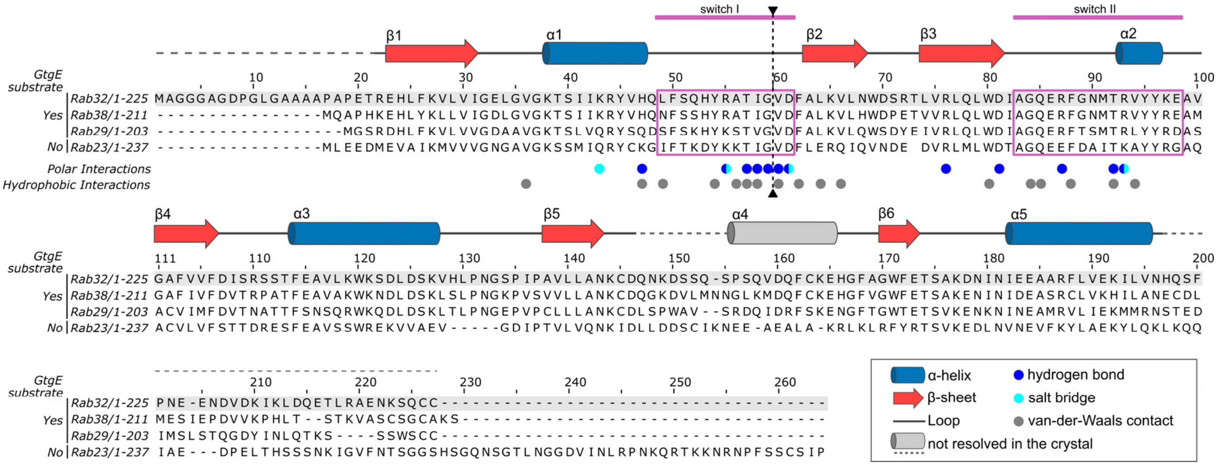


**Supplementary Fig. 1.** Sequence alignment of the GtgE-substrates Rab32, Rab38, and Rab29 in comparison to the non-GtgE substrate Rab23. Secondary structure annotations above the sequences refer to the Rab32:GDP:GtgE$_{C45A}$-complex. Blue or grey dots below the sequences indicate polar and hydrophobic interactions of Rab32 with GtgE in the complex structure. Magenta: switch regions; black arrow and dashed line: GtgE cleavage site in Rab32, Rab38, and Rab29.

*Supplementary Figure 2*

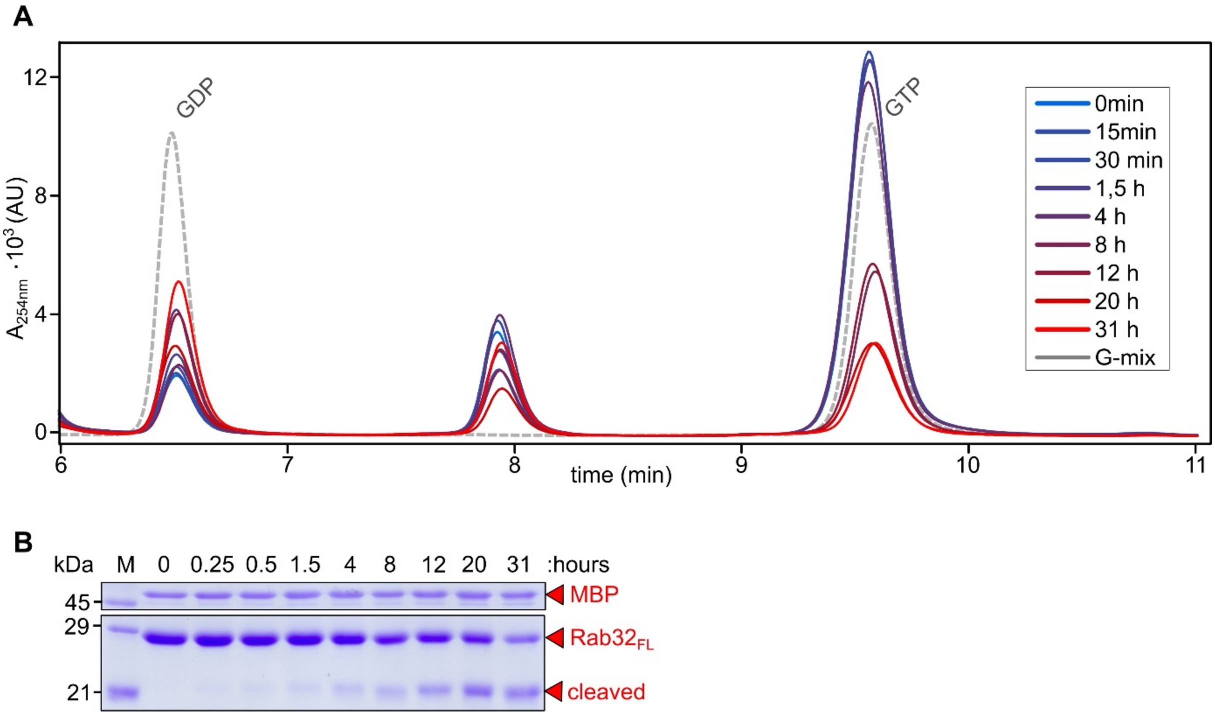


**Supplementary Fig. 2.** GTP-hydrolysis and proteolytic cleavage of Rab32:GTP.

(**A**) Intrinsic GTP-hydrolysis activity of Rab32. Time-dependent GTP-to-GDP-hydrolysis by Rab32 loaded with GTP was monitored using ion-pairing reversed phase chromatographic separation of GDP and GTP. Integrated peaks were used for the quantification of nucleotide content. The rate constant of GTP-hydrolysis was determined from single exponential fitting of the GTP-conversion with time (Fig. 1C). G-mix contains GDP and GTP as chromatographic references.

(**B**) Time-dependent GtgE-mediated (8 nM) cleavage of Rab32:GTP (8 µM final) in a SDS-PAGE based gel shift assay stained with Coomassie. MBP was used as internal standard for quantification. The observed rate constant for proteolysis was obtained from time-dependent single exponential fit of the $Rab32_{FL}$ concentration. The Rab32 level was determined from the SDS-PAGE band intensity, which was analyzed densitometrically (Fig. 1C).

***Supplementary Figure 3***

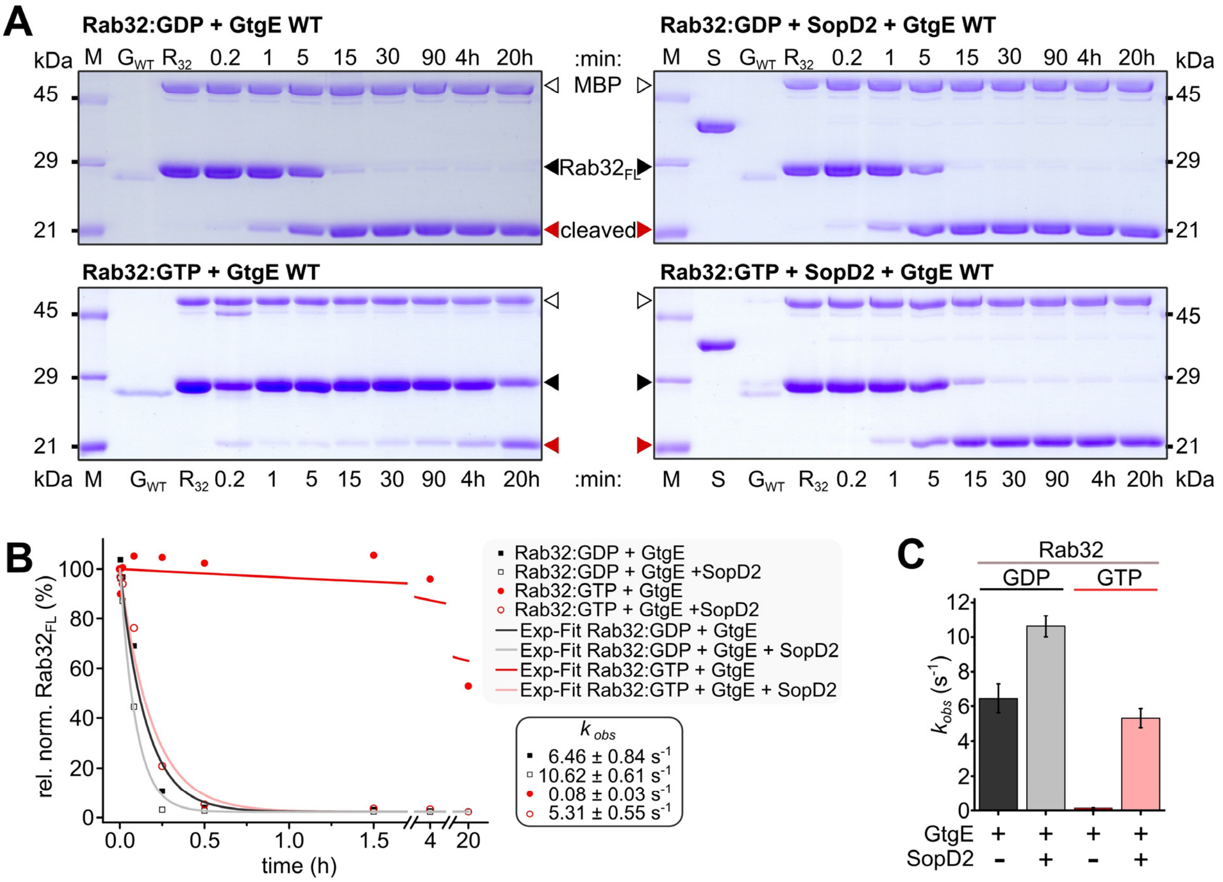


**Supplementary Fig. 3.** Dependence of Rab32-cleavage by GtgE on SopD2 mediated GTPase activation.

(**A**) SopD2 interference with GtgE mediated proteolytic Rab32 cleavage over time analysed by Coomassie-stained SDS-PAGE. Rab32$_{FL}$ (R$_{32}$;8 μM) was challenged with GtgE (G$_{WT}$; 8 nM) and/or SopD2 (S; 80 nM). White triangle: MBP standard; black arrow Rab32$_{FL}$; red arrow cleavage product Rab32$_{60\text{-}225}$.

(**B**) Densitometric quantification of the time-dependent decrease of Rab32$_{FL}$ bands from A. Signal intensity was normalized for the internal standard MBP. Data was fitted to a single exponential function to yield the observed rate constants ($k_{obs}$) (bottom right).

(**C**) Bar graph of the determined rate constant from B.

***Supplementary Figure 4***

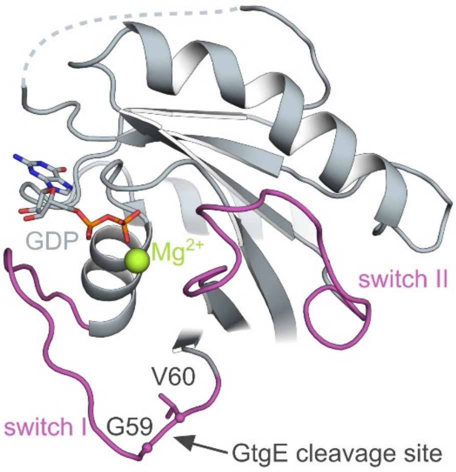


**Supplementary Fig. 4.** Structural context of the Rab32-cleavage site on the basis of the Rab32:GDP:GtgE$_{C45A}$-complex crystal structure. The GtgE cleavage site is highlighted in the ball and sticks representation of Rab32 residues Gly59 and Val60. Magenta loops: switch regions; sticks: GDP; green sphere: $Mg^{2+}$-ion.

*Supplementary Figure 5*

A

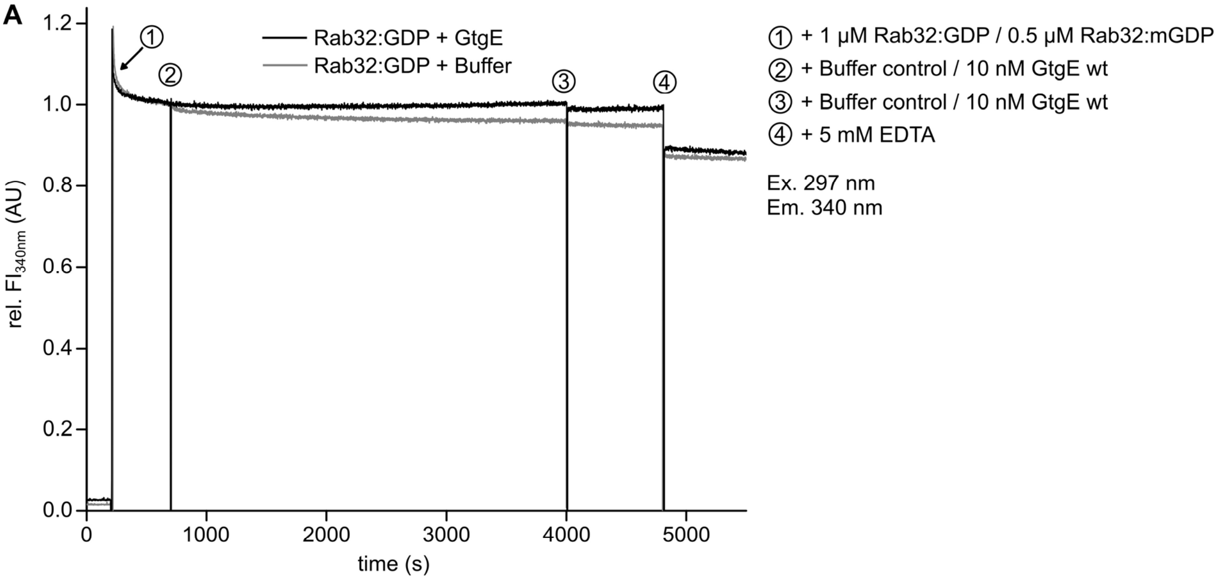


B

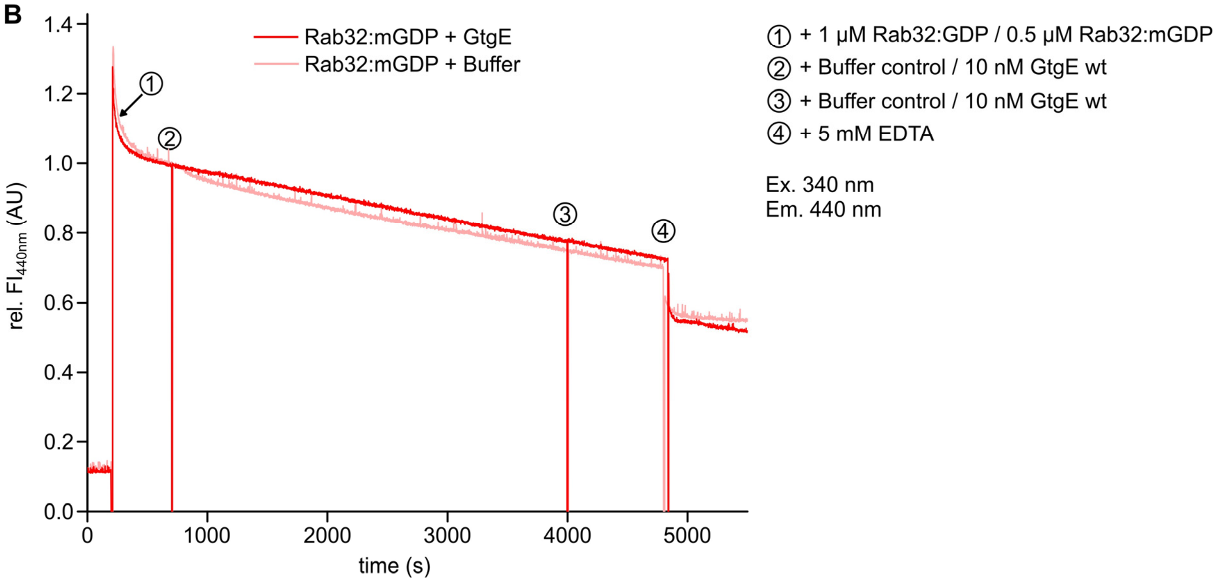


**Supplementary Fig. 5.** The GtgE-mediated Rab32-proteolysis cannot be monitored using established fluorescence methods. Rab32-proteolysis has been subjected to time dependent fluorescence experiments. Fluorescence intensity testing of GtgE-mediated Rab32-cleavage by Rab32 tryptophane fluorescence (**A**) or Rab32:mantGDP fluorescence (**B**). There is no significant difference of the fluorescence change in the absence or presence of GtgE, indicating that these assays are unsuitable for monitoring Rab32-proteolysis by GtgE.

*Supplementary Figure 6*

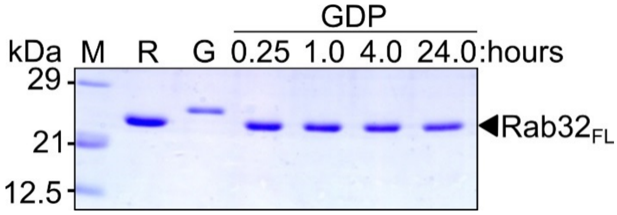


**Supplementary Fig. 6.** The GtgE-mutant GtgE$_{C45A}$ lacks proteolytic activity. Rab32$_{FL}$:GDP (R, 8 µM) was incubated with GtgE$_{C45A}$ (G, 8 nM) and samples were analyzed by Coomassie-stained SDS-PAGE at indicated time points. In contrast to wild type GtgE, the mutant GtgE$_{C45A}$ is proteolytically inactive as indicated by a lack of shift in Rab32$_{FL}$ molecular weight.

*Supplementary Figure 7*

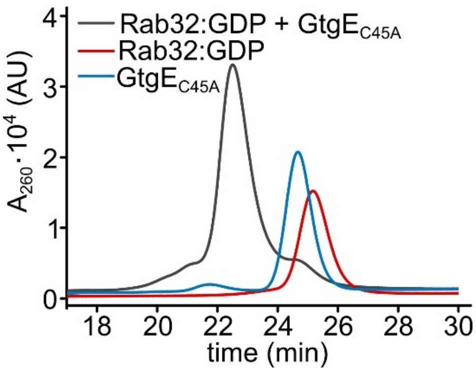


**Supplementary Fig. 7.** Complex formation of Rab32:GDP with GtgE$_{C45A}$ in analytical size exclusion chromatography. Rab32:GDP and GtgE$_{C45A}$ (8 µM each) were analyzed separately in SEC-buffer (supplemented with 10 µM GDP) as references. The decrease in elution time for the Rab32:GDP and GtgE$_{C45A}$ complex mixture indicates an increase in molecular weight relative to the individual proteins and demonstrates successful complex formation.

***Supplementary Figure 8***

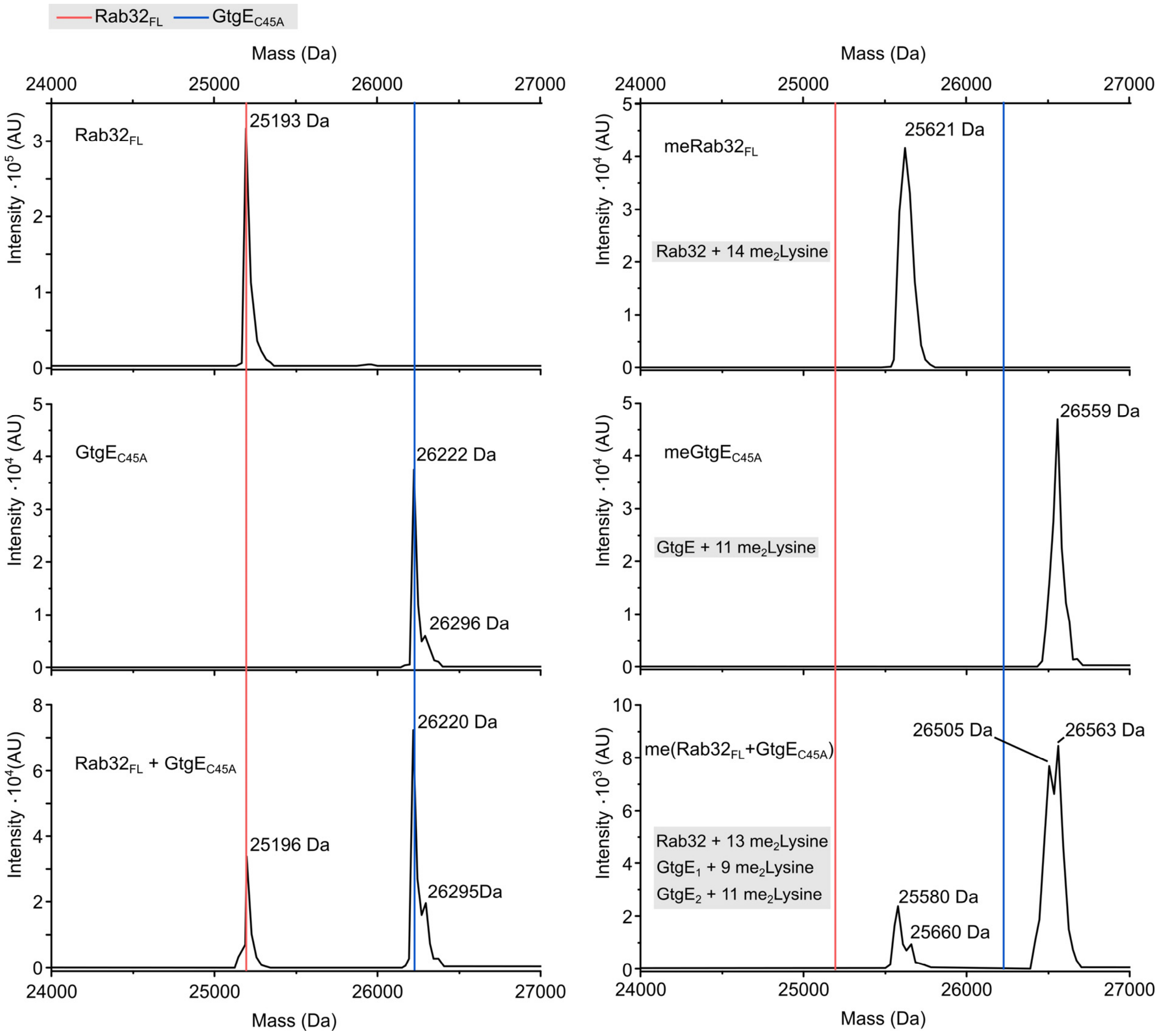


**Supplementary Fig. 8.** Mass spectrometry analysis of the methylated full length Rab32:GDP:GtgE$_{C45A}$ complex.

**Left panel**: top, Rab32$_{FL}$; middle, GtgE$_{C45A}$; bottom, 1:1 mixture of Rab32$_{FL}$ and GtgE$_{C45A}$.

**Right panel**: analysis of methylated (me) proteins (top, meRab32$_{FL}$; middle, meGtgE$_{C45A}$; bottom, 1:1 mixture of the preformed complex me(Rab32$_{FL}$:GtgE$_{C45A}$)). The red and blue lines indicate the theoretical masses of Rab32$_{FL}$ ($M_{cal(Rab32,FL)}$ = 25191 Da) and GtgE$_{C45A}$ ($M_{cal(GtgE,C45A)}$ = 26226 Da), respectively. Grey boxes: number of detected dimethylated lysines in the corresponding experiments.

*Supplementary Figure 9*

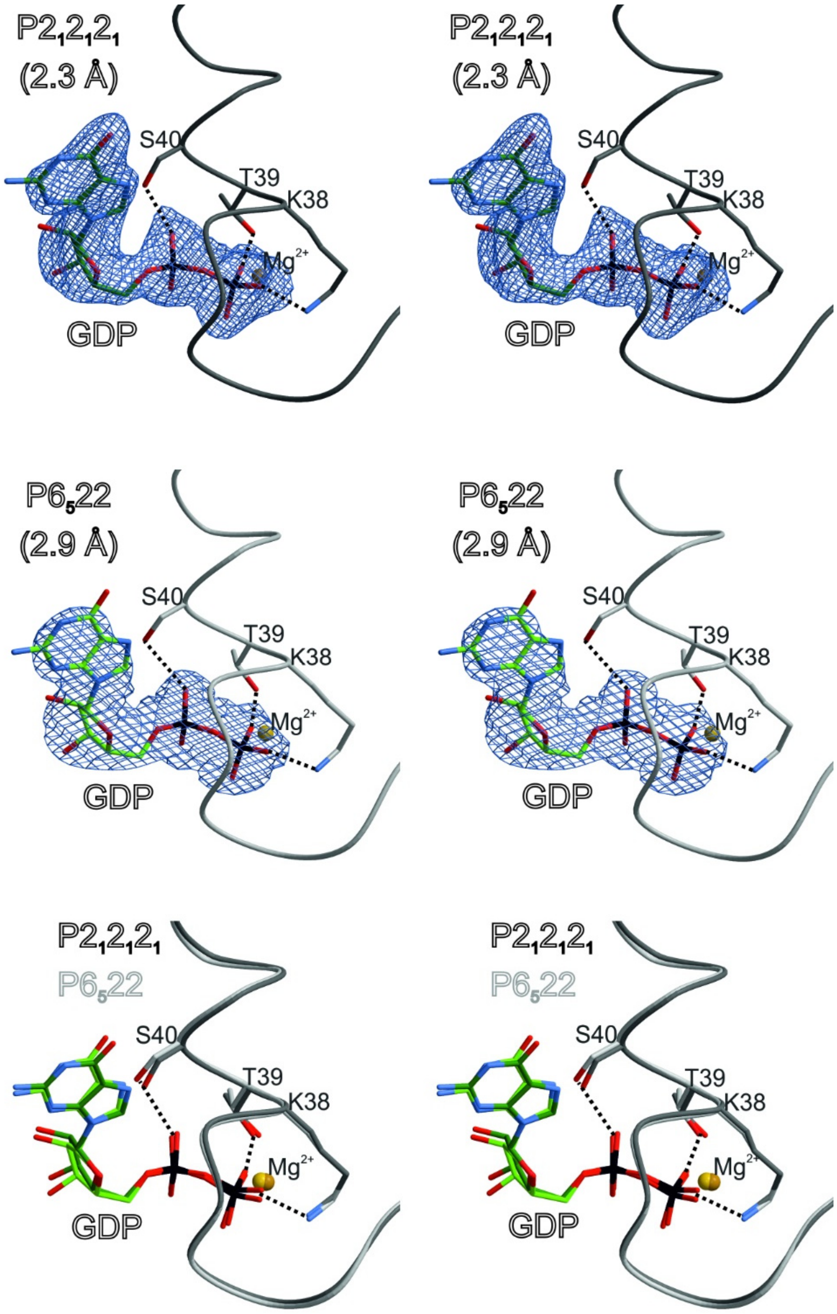


**Supplementary Fig. 9.** Comparison of the electron density of GDP in the full length and truncated Rab32:GtgE$_{C45A}$ complex structures depicted as stereo-view images.

Sections of the P-loop, the GDP-molecule and the magnesium ion of Rab32$_{18\text{-}201}$:GDP:GtgE$_{21\text{-}214,C45A}$ (top panel, PDB ID: 5OEC, space group P2$_1$2$_1$2$_1$, 2.3 Å resolution) and Rab32$_{FL}$:GDP:GtgE$_{FL,C45A}$ (middle panel, PDB ID: 5OED, space group P6$_5$22, 2.9 Å resolution), respectively. The 2F$_O$–F$_C$ electron-density maps depict the GDP nucleotides, which have been excluded prior to phase calculations (blue mesh, contour level at 1$\sigma$).

Structural superposition of both crystal structures revealing a close match of both complexes within this region (bottom panel).

*Supplementary Figure 10*

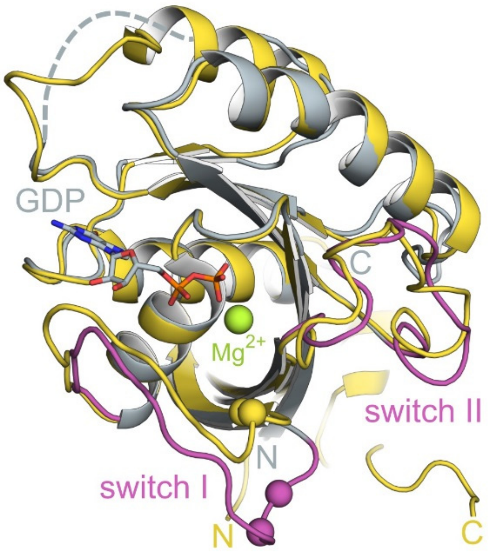


**Supplementary Fig. 10.** Structural superposition of Rab32 from the Rab32:GDP:GtgE$_{C45A}$ complex (grey) and Ypt1 from the yeast Ypt1:GDP:GDI complex (yellow, PDB ID: 1UKV [1]). The switch regions (indicated as magenta loops in Rab32) adopt a similar conformation that is clearly different from the active, GTP-bound conformations (see Fig. 3B). The switch structures are more reminiscent of but not equal to a GDI-bound state. Spheres: Cα-atoms of the GtgE cleavage site with the homologue positions also indicated in Ypt1; sticks: GDP; green sphere: $Mg^{2+}$-ion.

*Supplementary Figure 11*

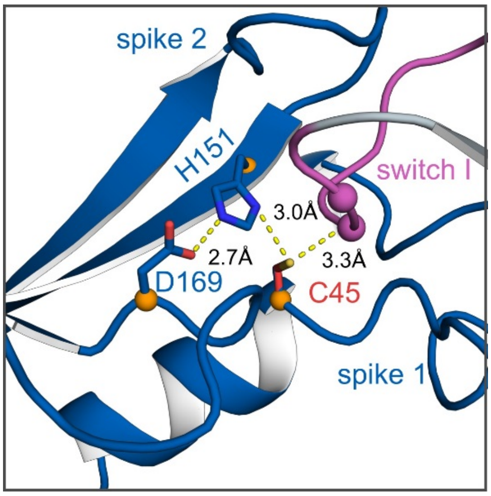


**Supplementary Fig. 11.** Model of the active catalytic triade in GtgE with the Rab32-substrate bound to the active site. The inactivating GtgE-mutation C45A$_G$ was reversed *in silico* using the PyMOL software [2], revealing a correctly aligned cysteine protease catalytic triad (*i.e.* constituted by C45$_G$, H151$_G$, and D169$_G$). Magenta loop: switch I of Rab32 from the Rab32:GDP:GtgE-complex; orange spheres and sticks: Cα-atoms and residues forming the catalytic triad, respectively; red sticks: *in silico* positioned cysteine at position 45 of GtgE in exchange for alanine; yellow dashed lines: distance measurements.

*Supplementary Figure 12*

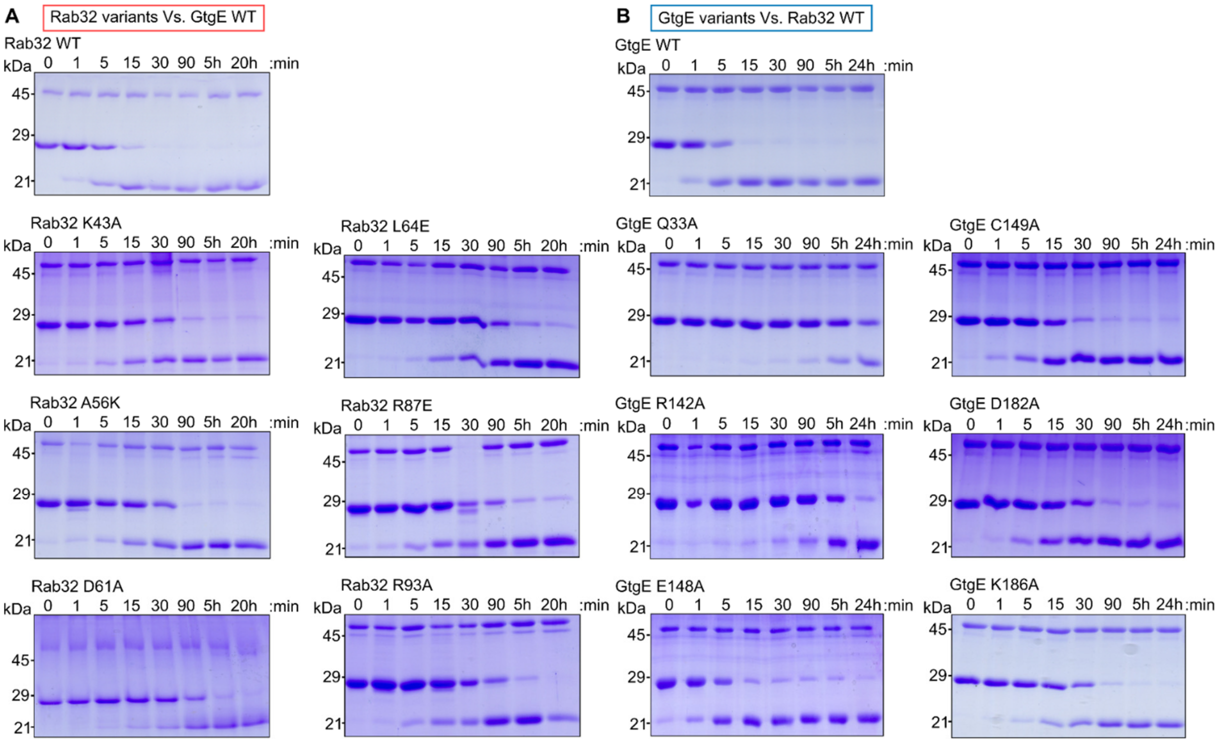


**Supplementary Fig. 12.** Mutational analysis of Rab32 and GtgE using a Coomassie-stained SDS-PAGE-based proteolytic Rab32 cleavage assay. These data form the basis for determining proteolytic rate constants as described in Supplementary Fig. 13.

(**A**) Proteolysis of Rab32:GDP and mutants thereof (8 μM) by wild type GtgE (8 nM).

(**B**) Proteolysis of Rab32:GDP (8 μM) with GtgE and mutants thereof (8 nM).

## *Supplementary Figure 13*

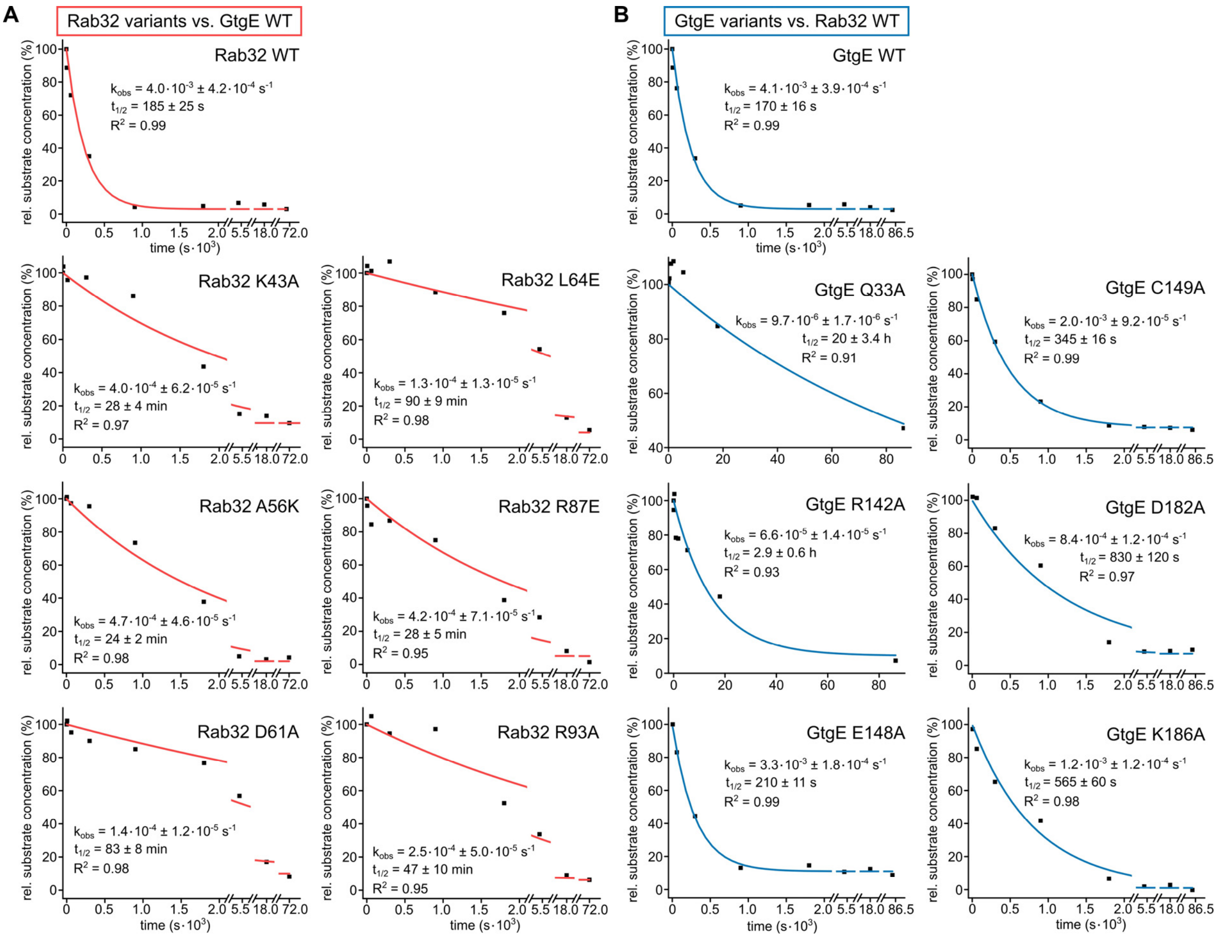


**Supplementary Fig. 13.** Determination of proteolytic rates GtgE for the Rab32-substrate and mutants thereof. Results are based on densitometric analysis of proteolytic Rab32-cleavage assays monitored by Coomassie-stained SDS-PAGE (Supplementary Fig. 12). The time-dependent decrease of Rab32$_{FL}$ was fitted to a single exponential function to yield the observed rate constants ($k_{obs}$). The $k_{obs}$-values have also been converted to half-lives ($t_{1/2}$) following the relationship $t_{1/2}$ = ln 2 / $k_{obs}$. Single exponential fits correspond to the experimental data very well as indicated by an $R^2$-value close to 1. Errors represent the standard deviation of the exponential fit.

(**A**) Rab32:GDP mutants (8 µM) and wild type incubated with wild type GtgE (8 nM) (red curves).

(**B**) GtgE mutants and wild type (both 8 nM final) were incubated with wild type Rab32:GDP (8 µM) (blue curves).

***Supplementary Figure 14***

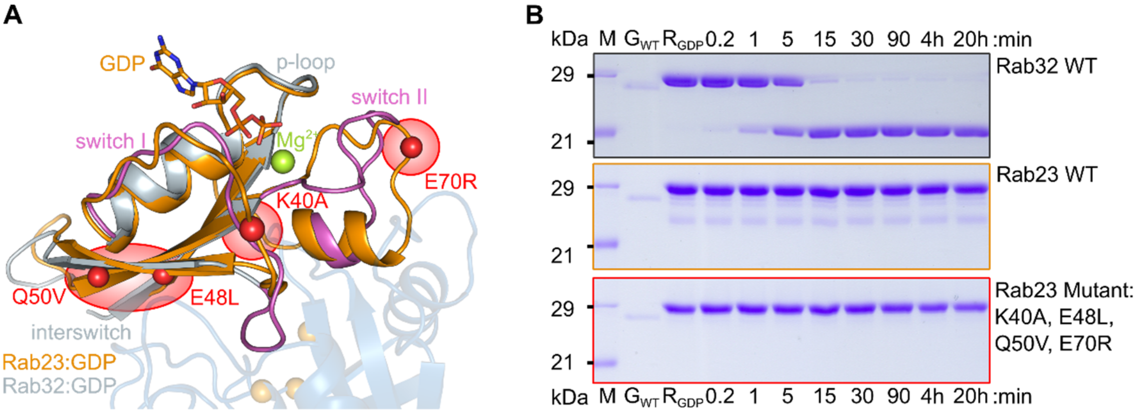


**Supplementary Fig. 14.** Exploration of the GtgE Rab-substrate specificity with a selected Rab23 mutant. Choice of mutation positions was guided by sequence alignment and interaction site analysis from the Rab32:GtgE-complex (see Supplementary Fig. 1).

(**A**) Structural superposition of the Rab23:GDP (orange) and Rab32:GDP (grey) in complex with GtgE$_{C45A}$ (blue, transparent) depicted as cartoon. Mutations in Rab23 to the corresponding amino acid from Rab32 were identified in three hot spots highlighted in red circles: switch I (K40A); interswitch (E48L; Q50V); switch II (E70R). Highlighted as red spheres: C$_{\alpha}$-atoms of respective mutations, sticks: GDP, magenta: switch regions, green sphere: $Mg^{2+}$-ion.

(**B**) Time dependent GtgE proteolysis gel shift assay of Rab32:GDP, Rab23:GDP, and Rab23:GDP mutant (K40A; E48L; Q50V; E70R). GtgE is unable to cleave Rab23 or Rab23-mutant containing respective Rab32-mutations, indicating that further structural determinants contribute to the Rab-specificity of the protease. Abbreviations: GtgE WT (G$_{WT}$, 8 nM), respective GDP-bound Rab (R$_{GDP}$, all 8 μM) without GtgE added.

*Supplementary Figure 15*

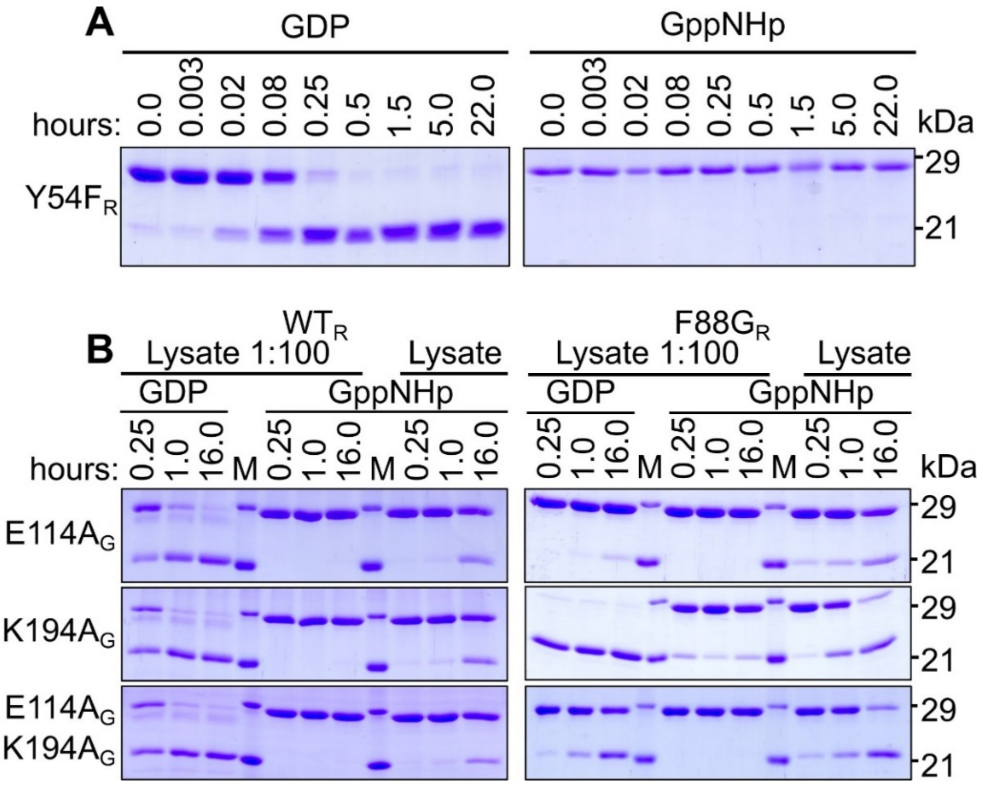


**Supplementary Fig. 15.** Qualitative analysis of the impact of mutations in potential nucleotide specificity hubs by gel shift activity assay.

(**A**) Significance of hub 2 mutant Y54F$_R$ loaded with GDP or GppNHp (compare with Fig. 5D).

(**B**) Significance of hub 3 mutants: Rab F88G loaded with GDP or GppNHp was treated with cleared *E. coli* lysate (or 1:100 diluted lysate) with overexpressed GtgE E114A, K194A, or the double mutant over time, respectively (see Fig. 5F).

***Supplementary Figure 16***

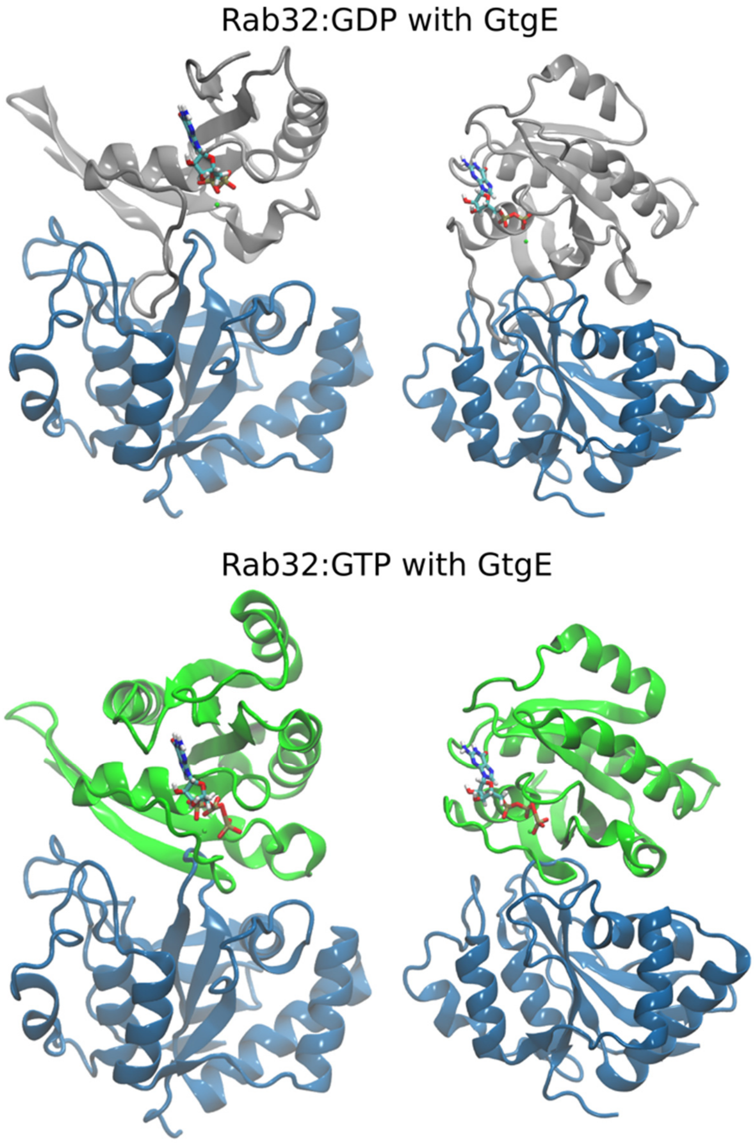


**Supplementary Fig. 16.** Models of GDP-bound (top panel) and a hypothetical GTP-bound Rab32 (bottom panel) in complex with wild type GtgE as starting points for atomistic molecular dynamics simulations. The GDP-bound Rab:GtgE complex is constituted based on the presented crystal structure from this work. The hypothetical GTP-bound complex structure is based on the active state Rab32 in complex with GppCH$_2$p and its effector binding domain VARP-ANKRD1 (PDB ID: 4CYM). Only the hypothetic Rab32:GTP:GtgE results in steric conflicts along the protein-protein interface particularly in the Rab switch I region with the spike 2 loop in GtgE. Both complexes are additionally shown 45° tilted on the y-axis.

***Supplementary Figure 17***

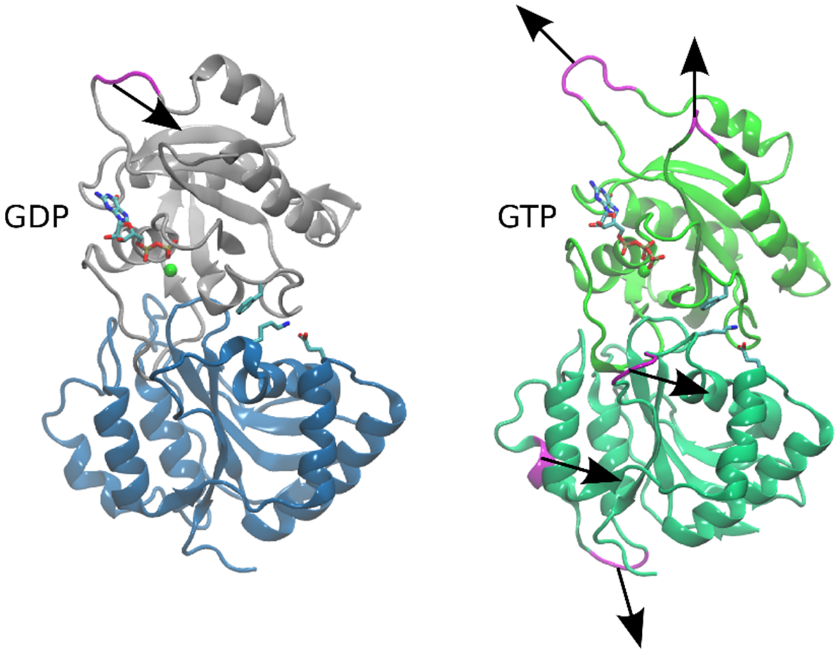


**Supplementary Fig. 17.** Principal component analysis of the Rab32:GtgE-complex in two different activity states. The figure shows the first modes of the principal component analysis of the Rab32:GDP:GtgE-complex (left) and the putative Rab32:GTP:GtgE-complex (right). While Rab32 (grey) of the GDP-complex only shows a single movement of a loop structure (purple), the GTP-complex reveals movements of two loop structures in Rab32 (light green) and three movements in GtgE (dark green). Interestingly, most of the movements of the GTP-complex point away from the interaction site of Rab32 and GtgE, while the movement of the GDP-complex points towards the interaction site of both domains. The diametral protein movements in the GTP-complex are supporting the complex dissociation for active Rab32:GTP, consistent with the strict GDP-state preference of GtgE for Rab32. The normal modes shown here, indicated by black arrows, comprise *ca*. 74% (GDP-complex) and *ca*. 77 % (GTP-complex) of their overall global motion.

***Supplementary Figure 18***

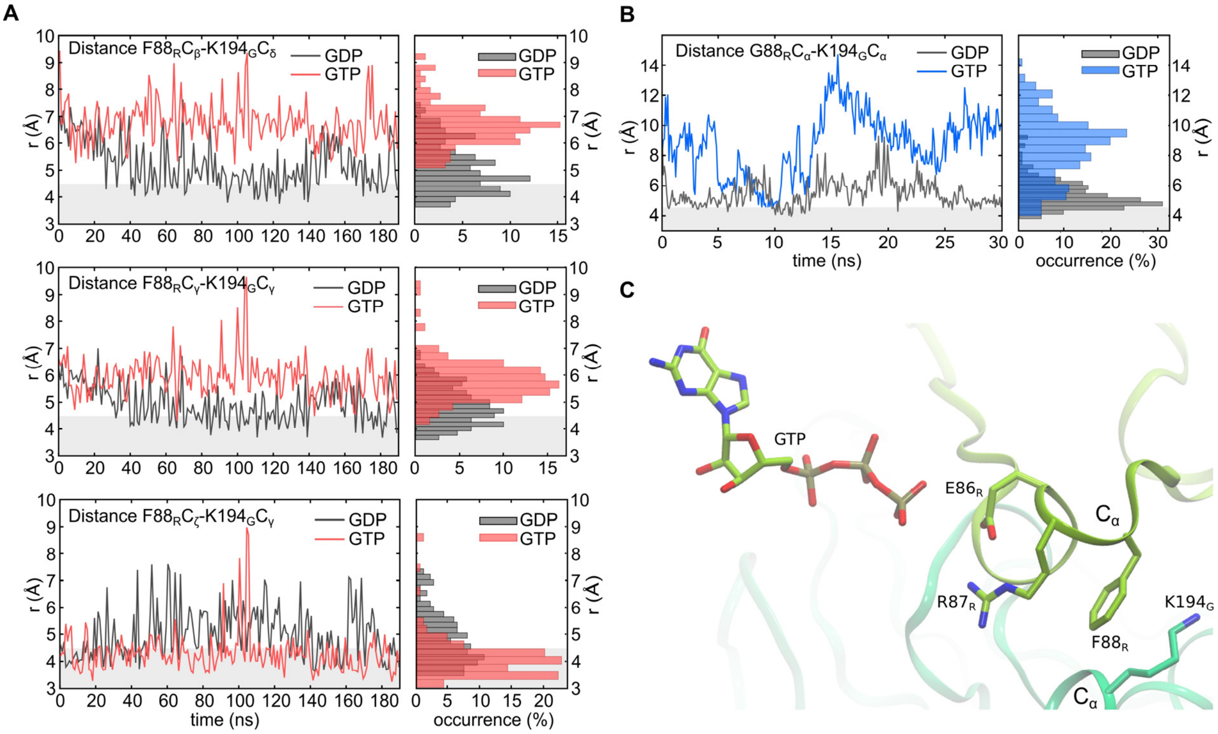


**Supplementary Fig. 18.** Selected atom pair distances in a GDP-bound and hypothetical GTP-bound Rab32:GtgE-complex from MD-simulations (left panels), and respective distance occurrences shown as histograms (right panels). The distance threshold for van-der-Waals interactions (<4.5 Å) is marked as grey shaded area.

(**A**) The F88$_R$ may form a decisive element for the GDP-state preference of GtgE toward Rab32. Specific atom-pair distances are shown for F88$_R$ of Rab32 and K194$_G$ GtgE (*top panel*: F88$_R$C$_\beta$-K194$_G$C$_\delta$, *middle*: F88$_R$C$_\gamma$-K194$_G$C$_\gamma$, *bottom*: F88$_R$C$_\zeta$-K194$_G$C$_\gamma$) from MD-simulations of the GDP- (in black) and GTP-bound (in red) Rab32:GtgE-complexes (see Fig. 5B right panels). A hydrophobic interaction between F88$_R$ and K194$_G$ is indicated based on all traces in the GDP-bound complex (black), whereas the hypothetic GTP-bound complex leads to an increase in the distance between F88$_R$C$_\beta$-K194$_G$C$_\delta$ and F88$_R$C$_\gamma$-K194$_G$C$_\gamma$, but not between F88$_R$C$_\zeta$-K194$_G$C$_\gamma$ (in red). This suggests that the van-der-Waals interactions between Rab32 and GtgE are decreased in the hypothetical GTP-state, which could lead to dissociation of GtgE from Rab32.

(**B**) Atom-pair distances in the F88G$_R$ mutant based on MD simulations of the GDP- (in black) and GTP-bound (in red) Rab32:GtgE-complexes. In F88G$_R$, a higher population of G88$_R$-K194$_G$ interactions (<4.5 Å) are formed relative to the wild type (Fig. 5E).

(**C**) Structural representation of the MD simulation of the hypothetical GTP-bound Rab32:GtgE-complex after 190 ns. Highlighted in sticks: hub 1 residue E86$_R$ with R87$_R$ forming an ion pair (see Fig. 5A and Fig. 5F, top) and the hub 3 resides F88$_R$ and K194$_G$ with an distance increase of the C$_\alpha$-atoms (see Fig. 5E, bottom).

## Supplementary Tables

### *Supplementary Table 1.*

Crystallographic statistics of the full length and truncated Rab32:GDP:$GtgE_{C45A}$-complex.

| | | **Rab32 (18-201):GDP-GtgE (21-214) C45A** | **Rab32:GDP-GtgE C45A** |
|---|---|---|---|
| <u>Crystal parameters</u> | | | |
| Space group | | $P2_12_12_1$ | $P6_522$ |
| Cell constants (Å) | | a = 47.8 , b = 67.0 | a = b = 67.4 |
| | | c = 111.1 | c = 427.3 |
| Copies in asym. unit | | 1 | 1 |
| <u>Data collection & processing</u> | | | |
| X-ray source | | SLS, X06DA | SLS, X06DA |
| Wavelength (Å) | | 1.0 | 1.0 |
| Resolution range (Å)[a] | | 50-2.3 (2.4-2.3) | 50-2.9 (3.0-2.9) |
| No. observations | | 82247 | 69825 |
| No. unique observations | | 16142 | 13564 |
| Completeness (%)[a] | | 97.4 (93.0) | 96.8 (98.1) |
| $R_{merge}$ (%)[a,b] | | 8.2 (52.3) | 6.2 (53.2) |
| $CC_{1/2}$ (%)[a] | | 99.7 (89.6) | 99.9 (83.4) |
| I/σ (I)[a] | | 13.8 (3.6) | 18.9 (2.9) |
| <u>Refinement</u> | | | |
| Resolution range (Å) | | 15-2.3 | 15-2.9 |
| No. refl. working set | | 16068 | 13425 |
| No. refl. test set | | 1603 | 1343 |
| No. non hydrogen (protein) | | 2838 | 2906 |
| No. of ligand atoms | GDP | 28 | 28 |
| | Mg | 1 | 1 |
| Water molecules | | 55 | 13 |
| $R_{work}/R_{free}$ (%)[c] | | 20.8 / 24.5 | 20.1 / 24.0 |
| r.m.s.d. bond (Å) / (°)[d] | | 0.002 / 0.435 | 0.004 / 0.527 |
| Average B-factor ($Å^2$) | Protein | 56.6 | 85.2 |
| | GDP; Mg | 60.2 | 114.5 |
| | Water | 42.7 | 57.1 |
| Ramachandran Plot (%)[e] | | 97.3 / 2.4 / 0.3 | 97.7 / 2.0 / 0.3 |

[a] The values in parentheses of resolution range, completeness, $R_{merge}$, $CC_{1/2}$ and I/σ (I) correspond to the last resolution shell. [b] $R_{merge}(I) = \sum_{hkl}\sum_j |[I(hkl)_j - I(hkl)]| / \sum_{hkl} I_{hkl}$, where $I(hkl)_j$ is the measurement of the intensity of reflection *hkl* and <I(*hkl*)> is the average intensity. [c] $R = \sum_{hkl} |\,|F_{obs}| - |F_{calc}|\,| / \sum_{hkl} |F_{obs}|$, where $R_{free}$ is calculated without a sigma cut off for a randomly chosen 5% of reflections, which were not used for structure refinement, and $R_{work}$ is calculated for the remaining reflections. [d] Deviations from ideal bond lengths/angles. [e] Number of residues in favored region / allowed region / outlier region.

***Supplementary Table 2.***

Molecular interactions in the Rab32:GDP:GtgE-complex between $Rab32_{18\text{-}201}$:GDP and $GtgE_{21\text{-}214,\ C45A}$ (Fig. 3E). (**A**) Polar interactions including salt bridges with the respective atom distances of interacting atom pairs. (**B**) Hydrophobic interactions shown as in A.

**A**

| Polar Interactions | | |
|---|---|---|
| **Rab32** | **Distance (Å)** | **$GtgE_{C45A}$** |
| G59 (O) | **2.44** | Q33 (NE2) |
| V60 (O) | **2.62** | S144 (OG) |
| T57 (O) | **2.67** | L150 (N) |
| D61 (O) | **2.70** | G146 (N) |
| D81 (N) | **2.75** | G146 (O) |
| D61 (OD1) | **2.76** | L145 (N) |
| T57 (OG1) | **2.88** | G80 (N) |
| R76 (NE) | **2.89** | N39 (OD1) |
| D61 (OD2) | **2.92** | R142 (NH2) |
| R76 (NH2) | **2.93** | T37 (O) |
| H47 (NE2) | **3.03** | I77 (O) |
| D61 (OD1) | **3.10** | R142 (NH1) |
| R55 (NH2) | **3.14** | E79 (OE2) |
| I58 (N) | **3.14** | I81 (O) |
| R55 (NH1) | **3.22** | D82 (OD2) |
| R93 (NE) | **3.23** | D182 (OD1) |
| G59 (N) | **3.24** | L150 (O) |
| R93 (NH2) | **3.27** | D182 (OD1) |
| I58 (O) | **3.29** | I81 (N) |
| G59 (O) | **3.36** | A45 (N) |
| V60 (O) | **3.41** | C149 (SG) |
| R87 (NH1) | **3.42** | K194 (O) |
| R93 (NH2) | **3.51** | S179 (OG) |
| D61 (O) | **3.52** | S147 (N) |
| D81 (O) | **3.68** | S147 (N) |
| V60 (N) | **3.70** | N43 (OD1) |
| D61 (O) | **3.80** | L145 (N) |

| Salt Bridges | | |
|---|---|---|
| **Rab32** | **Distance (Å)** | **$GtgE_{C45A}$** |
| K43 (NZ) | **4.00** | E148 (OE1) |
| K43 (NZ) | **3.69** | E148 (OE2) |
| R55 (NE) | **3.34** | E79 (OE2) |
| R55 (NH1) | **3.22** | D82 (OD2) |
| R55 (NH2) | **3.14** | E79 (OE2) |
| R93 (NE) | **3.23** | D182 (OD1) |
| R93 (NE) | **3.92** | D182 (OD2) |
| R93 (NH2) | **3.27** | D182 (OD1) |
| R93 (NH2) | **3.97** | D182 (OD2) |
| D61 (OD1) | **3.55** | R142 (NH2) |
| D61 (OD1) | **3.10** | R142 (NH1) |
| D61 (OD2) | **2.92** | R142 (NH2) |
| D61 (OD2) | **3.86** | R142 (NH1) |

**B**

| Hydrophobic Interactions | | |
|---|---|---|
| **Rab32** | **Distance (Å)** | **$GtgE_{C45A}$** |
| L64 | **3.5** | F34 |
| F62 | **3.5** | M171 |
| V94 | **3.7** | L145 |
| V60 | **3.8** | L41 |
| W80 | **3.8** | L145 |
| L64 | **3.9** | L41 |
| I58 | **3.9** | W46 |
| H47 | **3.9** | L78 |
| I58 | **3.9** | I81 |
| T92 | **3.9** | D185 |
| I58 | **3.9** | I202 |
| I58 | **4.0** | C86 |
| L49 | **4.1** | L78 |
| A56 | **4.1** | I83 |
| I58 | **4.1** | I141 |
| V94 | **4.1** | D185 |
| G59* | **4.2*** | A45* |
| I58 | **4.2** | L150 |
| I58 | **4.2** | A152 |
| F88 | **4.2** | K194 |
| Q85 | **4.2** | Y195 |
| V66 | **4.3** | N39 |
| T57 | **4.3** | N76 |
| V60 | **4.3** | M171 |
| T36 | **4.4** | E148 |
| Y54 | **4.4** | E148 |
| G84 | **4.4** | Y195 |
| [ V60 | 4.5 | N43 |
| T36 | 4.5 | E148 |
| A56 | 4.5 | C149 |
| D61 | 4.5 | H151 |
| V94 | 4.5 | K186 |
| F88 | 4.5 | Y195 ] |

* Interaction not shown in Fig. 3E due to mutation in GtgE C45A

[ ] potential Interactions with a distance of 4.5 Å not shown in Fig. 3E (bottom)

***Supplementary Table 3.***

Kinetic parameters of GtgE-activity for Rab32:GDP. Data are calculated from conversion rates of $Rab32_{FL}$ bands in a densitometric analysis from Coomassie-stained SDS-PAGE assays (Supplementary Fig. 12, 13).

| **GtgE** | **Rab32** | $k_{cat}/K_M$ **($s^{-1}M^{-1}$)** | **Activity relative to WT (%)** |
|---|---|---|---|
| WT | WT | $5.1 \times 10^5 \pm 4.9 \times 10^4$ | 100.0 |
| Q33A | WT | $1.2 \times 10^3 \pm 2.1 \times 10^2$ | 0.2 |
| R142A | WT | $8.3 \times 10^3 \pm 1.8 \times 10^3$ | 1.6 |
| E148A | WT | $4.2 \times 10^5 \pm 2.2 \times 10^4$ | 82.0 |
| C149A | WT | $2.5 \times 10^5 \pm 1.2 \times 10^4$ | 49.3 |
| D182A | WT | $1.0 \times 10^5 \pm 1.5 \times 10^4$ | 20.5 |
| K186A | WT | $1.5 \times 10^5 \pm 1.5 \times 10^4$ | 30.0 |
| WT | K43A | $5.1 \times 10^4 \pm 7.8 \times 10^3$ | 10.0 |
| WT | A56K | $6.1 \times 10^4 \pm 6.8 \times 10^3$ | 12.0 |
| WT | D61A | $1.7 \times 10^4 \pm 1.6 \times 10^3$ | 3.4 |
| WT | L64E | $1.6 \times 10^4 \pm 1.7 \times 10^3$ | 3.2 |
| WT | R87E | $3.1 \times 10^4 \pm 6.2 \times 10^3$ | 6.0 |
| WT | R93A | $5.2 \times 10^4 \pm 8.9 \times 10^3$ | 10.2 |

**Supplementary References**

1. Rak A*, et al.* Structure of Rab GDP-dissociation inhibitor in complex with prenylated Ypt1 GTPase. *Science* **302**, 646-650 (2003).

2. Schrodinger L. The PyMOL Molecular Graphics System, Version 1.8. (2015).
